# A survey of paediatric difficult peripheral intravenous access in the emergency department and use of point‐of‐care ultrasound

**DOI:** 10.1002/ajum.12353

**Published:** 2023-07-13

**Authors:** Clayton Lam, Lucy Dunstan, Amy Sweeny, Stuart Watkins, Shane George, Peter J. Snelling

**Affiliations:** ^1^ Department of Emergency Medicine Gold Coast University Hospital Southport Queensland Australia; ^2^ School of Medicine and Dentistry Griffith University Southport Queensland Australia; ^3^ Children's Critical Care Unit Gold Coast University Hospital Southport Queensland Australia; ^4^ Menzies Health Institute Queensland Griffith University Southport Queensland Australia; ^5^ Sonography Innovation and Research (Sonar) Group Southport Queensland Australia; ^6^ Child Health Research Centre University of Queensland South Brisbane Queensland Australia

**Keywords:** difficult intravenous access, education, emergency medicine, paediatric, peripheral intravenous catheter, point‐of‐care ultrasound, ultrasonography

## Abstract

**Introduction/Purpose:**

Peripheral intravenous catheter (PIVC) insertion can be challenging in children, with point‐of‐care ultrasound (POCUS) known to increase success rates. The objective of this study was to survey how emergency department (ED) clinicians identify and escalate paediatric patients with difficult intravenous access (DIVA), specifically the use of POCUS.

**Methods:**

This cross‐sectional study was conducted in an Australian academic mixed ED that surveyed resident medical officers (RMOs), registrars, consultants and senior paediatric nurses. A 15 multiple‐choice questionnaire evaluated clinicians experience with paediatric PIVC insertion, approach to identifying and managing DIVA and the use of POCUS or other adjuncts.

**Results:**

Eighty clinicians (34.2% response rate) completed the survey. Poor vein palpability was rated the highest predictor of DIVA. Of the respondents, 19 consultants (86.4%), 28 registrars (90.3%) and 16 RMOs (64.0%) used POCUS as an adjunct for paediatric DIVA patients but 16 consultants (72.8%), 21 registrars (67.8%) and 20 RMOs (80.0%) would use this less than 25% of the time in clinical practice.

**Discussion:**

This survey suggests more clinicians to prefer using objective factors when identifying paediatric DIVA patients, rather than subjectively using gestalt, which relies on clinician experience. Whilst clearly recognised as a useful tool in our study, POCUS was used infrequently for paediatric DIVA patients.

**Conclusions:**

There is currently no consistent process for the identification and escalation of paediatric DIVA patients, including the use of adjuncts such as POCUS. Clinician awareness for these issues should be addressed, which should include the development of guidelines and clinician training in POCUS for PIVC insertion in children.

## Introduction

Paediatric peripheral intravenous catheter (PIVC) insertion in the emergency departments (ED) can be a challenging procedure in children, with first attempt success rates reported to be around 40–50%.^1^, ^2^ Repeated attempts cause additional pain for the child, which can lead to psychological trauma and delays in medical treatment.^3^, ^4^ Failed attempts at PIVC insertion are multi‐factorial, including operator inexperience and patient characteristics that convey difficult intravenous access (DIVA).^5^

The early identification of children with DIVA has been shown to avoid multiple failed PIVC insertion attempts.^6^ Once identified, escalation measures can be taken to increase the likelihood of success with the minimum number of attempts. This includes escalation to a more experienced operator or use of an adjunct, such as transillumination, infrared or point‐of‐care ultrasound (POCUS).^7^, ^8^, ^9^, ^10^ In particular, the use of POCUS can improve paediatric PIVC insertion success rates, especially in patients with DIVA, with the use of a single operator, dynamic, short‐axis technique.^10^

Multiple studies have identified various objective and subjective factors that can assist with the identification of potential paediatric DIVA patients. Clinical tools have been validated using three to five variables to produce an overall ‘DIVA score’ to help identify these children in the ED.^5^, ^11^ These variables include vein visibility, vein palpability, younger child age, past history of DIVA and failed attempts.^5^, ^11^ Additionally, clinician gestalt (intuition or ‘gut feeling’) combined with PIVC insertion experience has been demonstrated to correlate with predicting a clinician's likelihood of PIVC insertion success in adults and children.^12^, ^13^

However, while there are tools that are available that can assist with predicting children with DIVA and existing evidence that POCUS increases PIVC insertion success in these patients, there are few studies that have specifically assessed clinician awareness of these issues.^9^ The primary objectives of this study were to survey the perceived knowledge, attitude and approach of clinicians towards the identification and management of paediatric DIVA patients in the ED and to specifically evaluate their use of POCUS as an adjunct.

## Methods and materials

This was a cross‐sectional study that surveyed ED clinicians at a large academic hospital located in South East Queensland, Australia, conducted between September and October 2021. The Gold Coast University Hospital ED has a co‐located paediatric emergency medicine (PEM) department, with an annual census of around 28,000 children under 16 years of age in 2021. There are a range of opportunities for ED ultrasound training for PIVC insertion, including workshop held regularly throughout the year.^14^ This is available to clinicians of all levels who insert PIVCs, including nurses. Most resident medical officers (RMOs), who are trainees not on a specialist pathway, sporadically work within the PEM department, and registrars, who are trainees on a specialist pathway, have a dedicated 3‐ to 6‐month rotation. RMOs are typically supervised during paediatric insertion.

Participants were eligible for the study if they were working in the ED and were directly involved in either the assistance or insertion of PIVCs in children. The clinicians surveyed included RMOs, registrars, consultants (mixture of emergency physicians and those with concurrent PEM qualification) and senior paediatric nurses. Participation was voluntary, with consent implied when participants completed the survey.

The survey was developed as a structured questionnaire, comprising 15 multiple‐choice questions (Appendix S1). Core themes included their level of experience with paediatric PIVC insertion and their routine approach to the identification and management of patients with DIVA. It also explored the use of adjuncts, specifically focusing on the use of POCUS. The survey was electronically distributed *via* Survey Monkey® (SurveyMonkey Inc., San Mateo, California, USA), an anonymous, web‐based electronic survey. Survey responses were de‐identified to ensure that investigators were blinded to individual clinician results. Descriptive statistics were used to calculate frequencies and percentages.

### Ethics approval

The Gold Coast Hospital and Health Service Human Research Ethics Committee (EC00160) approved the study (LNR/2019/QGC/55041).

## Results

The survey was distributed to 234 clinicians, including 63 consultants (6 PEM qualified), 57 registrars (ED trainees), 106 RMOs and eight senior nurses. Eighty clinicians completed the survey (34.2% response rate), comprising 22 consultants (3 PEM qualified) (34.9% subgroup; 27.5% overall respondents), 31 registrars (54.4% subgroup; 38.8% overall respondents), 25 RMOs (23.6% subgroup; 31.1% overall respondents) and 2 senior nurses (25.0% subgroup; 2.5% overall respondents).

All the consultants (100.0%) and most of the registrars (87.0%) performed at least 50 paediatric PIVCs, whereas only a small portion of the RMOs (8.0%) had inserted more than 50 paediatric PIVCs. Most of the consultants (95.5%) and registrars (80.7%) were at least somewhat comfortable with paediatric PIVCs, compared to only 20.0% of the RMOs (Table 1).

**Table 1:** Emergency department clinician experience with paediatric PIVC insertion.

	All respondents (n = 80)^a^		Consultant (n = 22)		Registrar (n = 31)		RMO (n = 25)	
	n	%	n	%	n	%	n	%
Years in current role								
One	21	26.3	4	18.2	5	16.1	11	44.0
Two	14	17.5	1	4.5	5	16.1	8	32.0
Three	7	8.8	0	0.0	2	6.5	5	20.0
Four	7	8.8	0	0.0	6	19.4	1	4.0
Five or more	31	38.8	17	77.3	13	41.9	0	0.0
Number of paediatric PIVC insertions performed in career								
<10	12	15.0	0	0.0	0	0.0	12	48.0
10–50	16	20.0	0	0.0	4	12.9	11	44.0
51–100	20	25.0	5	22.7	14	45.2	1	4.0
>100	32	40.0	17	77.3	13	41.9	1	4.0
Comfortability with paediatric PIVC insertion								
Very uncomfortable	4	5.0	0	0.0	0	0.0	4	16.0
Somewhat uncomfortable	14	17.5	1	4.5	3	9.7	10	40.0
Neutral	9	11.3	0	0.0	3	9.7	6	24.0
Somewhat comfortable	31	38.8	11	50.0	14	45.2	5	20.0
Very comfortable	22	27.5	10	45.5	11	35.5	0	0.0

Abbreviations: PIVC, peripheral intravenous catheter; RMO, resident medical officer.

^a^ Includes two senior nurse respondents.

**Figure 1:**
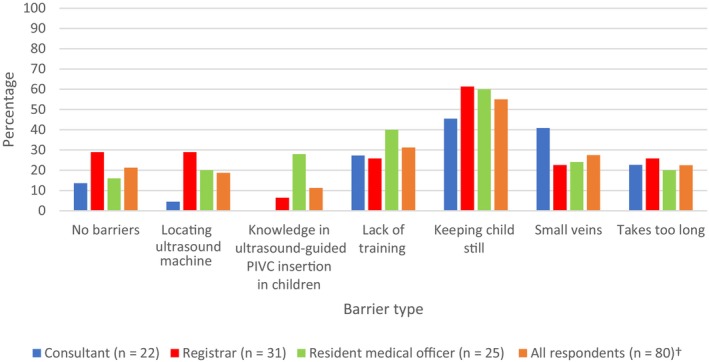
Barriers to using POCUS for paediatric patients with DIVA in the ED. ^†^Includes two senior nurse respondents. Abbreviations: DIVA, difficult intravenous access; ED, emergency department; PIVC, peripheral intravenous catheter; POCUS, point‐of‐care ultrasound.

When identifying potential paediatric DIVA patients, the highest proportion of clinicians of each type indicated that poor vein palpability was the best predictor (81.3%), followed by poor vein visibility (62.5%), known history of DIVA (61.3%), failed attempts (55.0%), child age (51.3%) and then clinician gestalt (37.5%; Figure 1). Only two clinicians (both consultants) had an awareness of existing paediatric DIVA scoring systems.

In terms of escalation, seven consultants (31.8%) and 22 registrars (71.0%) reported that at least two attempts were suitable prior to escalation to another operator with more experience. Also, 16 registrars (51.6%) and 16 RMOs (64.0%) would have one attempt prior to using an adjunct. However, 8 consultants (36.4%) would have two attempts prior to using an adjunct.

**Figure 2:**
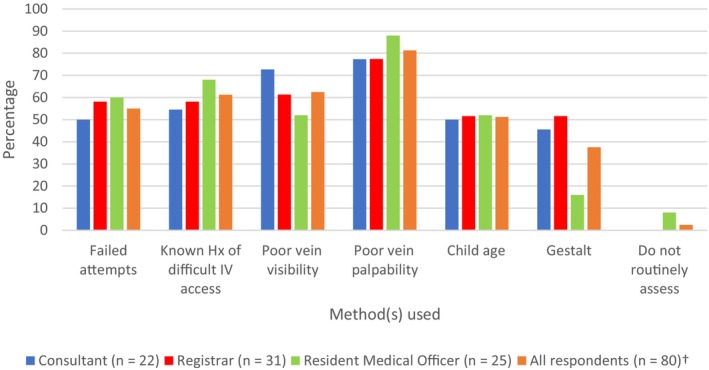
Identification of children with DIVA by ED clinicians. ^†^Includes two senior nurse respondents. Abbreviations: DIVA, difficult intravenous access; ED, emergency department; Hx, history; IV, intravenous.

Most of the survey respondents, including 19 consultants (86.4%), 28 registrars (90.3%) and 16 RMOs (64.0%), had used POCUS previously for PIVC insertions, and approximately half of each clinician type had used transillumination or vein‐location devices like infra‐red (Table 2). However, only around half of consultants (45.5%) and registrars (58.1%) used POCUS less than 25% of the time to assist PIVC insertion in paediatric DIVA patients, while almost half of the RMOs (48.0%) had never used POCUS in this context (Table 2). Overall, the majority of clinicians, including 14 consultants (80%), 26 registrars (83.9%) and 22 RMOs (88%), expressed interest in further training in POCUS for PIVC insertion in children. Around half of the total respondents (44, 55.0%) reported that the greatest barrier to using POCUS for paediatric PIVC insertion was stabilisation of the child's target insertion site during the procedure (Figure 2).

**Table 2:** Approach to the paediatric patient with DIVA requiring PIVC insertion by emergency department clinicians.

	All respondents (n = 80)		Consultant (n = 22)		Registrar (n = 31)		RMO (n = 25)	
	n	%	n	%	n	%	n	%
Immediate next step after patient confirmed to have DIVA								
Change operator (preference 1)	26	32.5	8	36.4	2	6.5	14	56.0
Change operator (preference 2)	42	52.5	9	40.9	22	71.0	11	44.0
Change operator (preference 3)	12	15.0	5	22.7	7	22.6	0	0.0
Use adjunct (preference 1)	48	60.0	11	50.0	27	87.1	10	40.0
Use adjunct (preference 2)	28	35.0	9	40.9	3	9.7	14	56.0
Use adjunct (preference 3)	4	5.0	2	9.1	1	3.2	1	4.0
Continue attempts (preference 1)	6	7.5	3	13.6	2	6.5	1	4.0
Continue attempts (preference 2)	10	12.5	4	18.2	6	19.4	0	0.0
Continue attempts (preference 3)	64	80.0	15	68.2	23	74.2	24	96.0
Attempts prior to escalation to another operator								
1	15	18.8	0	0.0	0	0.0	14	56.0
2	39	48.8	7	31.8	22	71.0	9	36.0
3	14	17.5	9	40.9	4	12.9	1	4.0
>3	7	8.8	4	18.2	3	9.7	0	0.0
Other	5	6.3	2	9.1	2	6.5	1	4.0
Attempts prior to using an adjunct								
1	37	46.3	4	18.2	16	51.6	16	64.0
2	24	30.0	8	36.4	10	32.3	6	24.0
3	4	5.0	3	13.6	1	3.2	0	0.0
>3	3	3.8	2	9.1	1	3.2	0	0.0
Other	12	15.0	5	22.7	3	9.7	3	12.0
Modification to next step if suspected DIVA patient (multiple responses accepted)								
Use adjunct on first attempt	43	53.8	11	50.0	22	71.0	9	36.0
Proceed with landmark attempt	25	31.3	10	45.5	11	35.5	3	12.0
Seek alternative operator	21	26.3	4	18.2	2	6.5	15	60.0
Adjuncts used for paediatric DIVA patients (multiple responses accepted)								
Ultrasound assisted	65	81.3	19	86.4	28	90.3	16	64.0
Vein location device	38	47.5	10	45.5	17	54.8	11	44.0
Transillumination	26	32.5	8	36.4	11	35.5	5	20.0
Knowledge of scoring systems								
Yes	2	2.5	2	9.1	0	0.0	0	0.0
No	78	97.5	20	90.9	31	100.0	25	100.0
Likelihood of ultrasound use in paediatric DIVA patients^a^								
Never	21	26.3	6	27.3	3	9.7	12	48.0
<25% of the time	38	47.5	10	45.5	18	58.1	8	32.0
25–50% of the time	8	10.0	3	13.6	3	9.7	2	8.0
More than 50% of the time	12	15.0	3	13.6	7	22.6	2	8.0
Other^a^	1	1.3	0	0.0	0	0.0	1	4.0
Further training in POCUS use								
Yes	64	80.0	14	63.6	26	83.9	22	88.0
No	16	20.0	8	36.4	5	16.1	3	12.0

Abbreviations: DIVA, difficult intravenous access; PIVC, peripheral intravenous catheter; POCUS, point‐of‐care ultrasound; RMO, resident medical officer.

^a^ Other free text response: Only after one attempt.

## Discussion

In this survey of ED clinicians' perceived understanding and attitudes towards PIVC insertion, there was no consistent approach to the identification and escalation of paediatric patients with DIVA, including the use of POCUS. Whilst nearly all surveyed clinicians were unfamiliar with validated paediatric DIVA scoring systems, more than half were able to identify a broad range of factors that predict DIVA in children (Figure 2). However, while recognised as a useful tool, POCUS was used infrequently for paediatric patients with DIVA.

The identification of a paediatric patient with DIVA remains inconsistent. Objective elements were rated more highly (*e.g*. vein palpability/visibility), while clinician gestalt was rated the lowest. To the contrary, studies have demonstrated that clinician gestalt combined with experience can rapidly and accurately identify patients with DIVA.^12^, ^13^ From our study, it may suggest that more clinicians prefer using objective factors when identifying paediatric DIVA patients, rather than subjectively using gestalt, which relies on clinician experience. While potentially more time consuming, implementation of a DIVA scoring system with objective variables could prompt less experienced operators to think about the level of difficulty and need for escalation prior to insertion attempt.

In this survey, the escalation of paediatric DIVA patients also varied according to clinician type. Deciding when and how a patient should be escalated was likely influenced by the differing opinions on what classified a DIVA patient. A standardised escalation pathway would help streamline this process, but should incorporate inserter competency to guide the number of attempts prior to escalation to a more experienced or different operator, or using an adjunct early like POCUS.^8^ Early escalation to advanced inserters after identification of a potential child with DIVA could potentially avoid multiple failed attempts.^6^, ^15^ However, escalation pathways should always consider the resources available when developing local policy, which include staff and POCUS availability.^16^

While clearly recognised as a useful tool in our study and having a strong evidence base, POCUS was used infrequently for paediatric DIVA patients.^10^ Point‐of‐care ultrasound is an additional skill that should be acquired once there is proficiency with landmark insertion of PIVCs in paediatric patients. It was highlighted in the survey responses that most consultants and registrars were comfortable with paediatric PIVC insertion as opposed to less than half of the RMOs, logically making consultants and registrars the main target for POCUS training for PIVC insertion in children with DIVA. Additionally, the most frequently reported barrier to its use was stabilisation of the child's target insertion site. Employing distraction and sedation during POCUS, where appropriate, may help increase successful PIVC insertions and increase its adoption.^10^ This is important as the use of POCUS can reduce the number of attempts required, which should then reduce psychological harm.^17^ Ideally, a guideline should be developed and adopted that covers all aspects of paediatric PIVC insertion, including early recognition of DIVA, analgesia and sedation, effective holding, escalation and use of POCUS.

### Limitations

There were various limitations to this study. Being a cross‐sectional study in a single centre, the results may not be generalisable to other settings. The response rate of 34.2% (total responses 80/342) was lower than anticipated and may have been biased towards clinicians working more regularly in PEM or were more familiar with PIVC insertion in children. Although there was a reasonable representation of each clinician subgroup, the survey did not have enough responses to make meaningful comment on nurses' practice, including their role in the use of POCUS for PIVC insertion in paediatric patients with DIVA. The strengths of this study were the broad range of ED clinicians surveyed from a large mixed centre and the novelty of evaluating current clinician understanding on this topic.

## Conclusion

In this single‐centre, cross‐sectional study of ED clinicians in an Australian academic mixed hospital, this study identified limited awareness and no consistent process for the identification and escalation of paediatric patients with DIVA, particularly with the use of POCUS. Although POCUS was reported to be the most popular adjunct for DIVA in children, it is currently underutilised. Additionally, this survey highlighted the need to focus on consultants and registrars for POCUS training, given they are the most comfortable with PIVC insertion in children. Lack of clinician awareness of these issues should be addressed, which should include the development of guidelines and clinician training in POCUS.

## Authorship statement

We acknowledge that (i) the authorship listing conforms with the journal's authorship policy, and (ii) that all authors are in agreement with the content of the submitted manuscript.

## Funding

This study was supported by grants from the Gold Coast Health Study, Education & Research Trust Account (SERTA, 2019) and the Emergency Medicine Foundation (EMTR‐150R32‐2019; Round 32, 2019).

## Conflict of interest

None to declare.

## Supporting information


**Appendix S1** Survey of paediatric peripheral intravenous catheter insertion in the emergency department Top of Form.
