# Supplementary material for: A survey of paediatric difficult peripheral intravenous access in the emergency department and use of point‐of‐care ultrasound
Source: Australas J Ultrasound Med. 2023 Jul 13;26(3):184–90. doi: 10.1002/ajum.12353 (PMC10493356; doi:10.1002/ajum.12353)
Supplement: Supplementary file 1 — Appendix S1 Survey of paediatric peripheral intravenous catheter insertion in the emergency department Top of Form. [file AJUM-26-184-s001.docx]

**Appendix 1: Survey of paediatric peripheral intravenous catheter insertion in the emergency department Top of Form**

**1. What is your current role in the emergency department?**

RMO

Registrar

Consultant (FACEM)

Consultant (PEM)

Nurse

**2. How many years in your current role?**

1

2

3

4

>5

**3. How many paediatric (<16yrs old) peripheral intravenous catheter insertions have you performed in your career?**

< 10

10 - 50

51 - 100

> 100

**4. What is your level of comfort when inserting peripheral intravenous catheters in children?**

Very UNcomfortable

Somewhat UNcomfortable

Neither UNcomfortable or comfortable

Somewhat comfortable

Very comfortable

**5. If you encounter a patient with difficult intravenous access, what is your order for immediate next step:**

Change operator 1 2 3

Use adjunct 1 2 3

Continue attempts without use of adjunct or changing operator 1 2 3

**6. If a paediatric patient is difficult to cannulate, how many attempts do you have prior to escalating to another operator?**

1

2

3

>3

Other (please specify)

**7. If a paediatric patient is difficult to cannulate, how many attempts do you have before using an adjunct?**

1

2

3

>3

Other (please specify)

**8. If you identify a patient as having the potential for difficult intravenous access, does this modify what you do next (select all that apply)?**

Seek alternative operator

Proceed with landmark attempt

Use of an adjunct with your first attempt (please specify )

**9. What adjuncts do you use for a difficult peripheral intravenous catheter insertion (select all that apply)?**

Transillumination

Vein location device

Ultrasound

Other (please specify)

**10. How do you identify patients with difficult intravenous access (select all that apply)?**

Failed attempts

Known history of difficult access

Poor visibility of veins

Poor palpability of veins

Age of child

Gestalt

I do not routinely assess children for difficult intravenous access

Other (please specify)

**11. Are you aware of any scoring systems or tools that identify difficult intravenous access in paediatric patients?**

No

Yes (Please specify scoring system you are aware of)

**12. How often would you use ultrasound to aid peripheral intravenous cannulation in children with difficult access?**

Never

< 25%

25 - 50%

> 50 %

 Only after a certain number of failed attempts with other methods (please specify)

**13. What do you perceive as barriers to using ultrasound for peripheral intravenous catheter insertion in children (select all that apply)?**

No barriers

Difficulty locating the ultrasound machine

Poor knowledge of how to use the ultrasound machine for peripheral intravenous catheter insertion

Lack of training in ultrasound guided vascular access for paediatric patients

Difficulty holding the patient still enough to use the ultrasound machine

Veins are too small and "fiddly"

Takes too long

Other (please specify)

**14. Are you aware of the benefits of ultrasound assisted peripheral intravenous catheter insertion for paediatric patients with difficult intravenous access?**

Extremely aware

Very aware

Somewhat aware

Not so aware

Not at all aware

**15. Would you be interested in more training in ultrasound use for peripheral intravenous access in children?**

Yes

No

Any other comments [Free text]

Bottom of Form
